# Denormalising tobacco at a Danish music festival

**DOI:** 10.18332/tpc/112672

**Published:** 2019-10-21

**Authors:** Maja K. Schjørring, Maria Stage, Andrea S. Glahn, Susan Andersen, Niels T. Kjær, Morten K. Grønbæk, Astrid Knudsen

**Affiliations:** 1 National Institute of Public Health, Copenhagen University, Copenhagen, Denmark; 2 The Danish Cancer Society, Copenhagen, Denmark

**Keywords:** tobacco industry, advocacy, denormalising, advertising and promotion, youth

## INTRODUCTION

Music festivals and similar arenas are used worldwide by the tobacco industry to promote tobacco to youth and to shape brand image and generate brand recognition^1,2^. The industry uses subtle tobacco promotion described as ‘below-the-line promotion’, such as handing out free cigarette samples or engaging in tobacco sponsorships^3,4^. Given the enormous impact of music on youth lifestyle and identity formation, music events are considered efficient arenas for tobacco promotion and marketing^1,2^. In Denmark, the tobacco advertising ban from 2008 is subject to various exemptions, among others allowing for a ‘neutral placement of tobacco’ at point-of-sale^5^. This exemption is utilised by the tobacco industry to market and promote tobacco. In spite of the ban on sponsorship, the industry provides festivals with funding and free cigarettes for resale, in exchange for exclusive agreements and promotion of tobacco products. A 2010 study of tobacco use at the largest Danish music festival, Roskilde Festival, found that 9% of never-smokers had consumed tobacco for the first time during the festival while 24% of those who had quit smoking, within the past year, relapsed into tobacco use during the festival^6^. The festival environment thus presents a great risk for youth smoking initiation and relapse.

This article describes the adoption and implementation of tobacco-free initiatives to end tobacco promotion and to denormalise tobacco use at the Danish music festival Strøm.

## THE CASE

Strøm is an electronic music organization based in Denmark. Strøm aims to stimulate and develop the electronic music scene both in Denmark and internationally through music events, educational activities, workshops and mainly targets children, adolescents and young adults. The largest event is the yearly Strøm Festival in the Copenhagen area. Each year, Strøm activities attract more than 15000 people, mainly adolescents and young adults aged 18–30 years (Christensen MS, Schjørring MK, unpublished data, 2015).

A 2015 survey of Strøm Festival found that 47% (n=602) of the festival audience had smoked a cigarette on the day or the day before going to the festival, indicating that tobacco played an important role for many of the festival participants (Christensen MS, Schjørring MK, unpublished data, 2015).

From its establishment in 2007, smoking and tobacco have been an integral part of the visual identity of Strøm. Tobacco products were consistently visible and thus indirectly promoted in social media posts and visual communication material related to the festival. However, in 2014 Strøm decided to cease the indirect promotion and the sale of tobacco products. The decision was based on the role of Strøm as an important facilitator of educational activities for children and youth, which was not ethically compatible with promotion and sale of tobacco. Nonetheless, this was a difficult decision, considering the loss of funding from tobacco sponsorships and concerns about negative feedback from the audience.

To consolidate the decision to cease promotion and sale of tobacco among Danish stakeholders, Strøm initiated a partnership with the Danish Cancer Society, the National Institute of Public Health, and The Health and Care Administration of the Municipality of Copenhagen.

The aim of the partnership was to denormalise tobacco use and to inspire reflections about the presence of the tobacco industry in the music scene. To reach this goal the partnership focused on three key activities: 1) To create awareness among Strøm staff of the presence of tobacco and their role in indirectly promoting tobacco in the music scene, 2) To end exposure to tobacco marketing and to eliminate tobacco sponsorship in Strøm, and 3) To inspire a wider range of stakeholders in the music industry to reflect upon the relationship between tobacco and music with the hope to expand tobacco-free activities to other music organisations.

The initiatives were funded by the Municipality of Copenhagen, while the other partners contributed with specialist knowledge to qualify and share knowledge, since this was the first initiative of its kind. Strøm led the formation and implementation of the initiatives.

### Qualitative interviews with Strøm staff

The first initiative of the tobacco-free strategy consisted of qualitative interviews with employees and volunteers within the Strøm organisation (n=17). Most Strøm staff members also work in other positions in the Danish music scene. In the interviews, examples of direct and indirect tobacco promotion were shown to the respondents and debated subsequently.

Using the potential of the interview as an intervention, they aimed to create awareness among the employees and volunteers of the presence of tobacco and smoking in the music scene and to make them reflect on their own experience, attitudes and behaviour to smoking.

Several of the Strøm staff members interviewed considered smoking to be a socially accepted and integral part of the music scene and associated it with positive social situations.

Interviewees mentioned that they had experienced tobacco marketing at music festivals and at cultural events in general.

*‘I volunteered in the backstage area at a danish music festival one year, and BAT [British American Tobacco] sponsored all of the area. A few girls were to stand in a bar made of an old minivan and told to smoke or pretend to smoke. We were told that it almost didn’t matter whether we knew the bar menu or not, the pivotal thing was to know everything about their cigarettes and put them on the counter in a certain way when people bought a drink, small subtle triggers like that.’* (Strøm worker)

Staff members expressed a clear understanding of the financial dependency on the tobacco industry for music organisations.

*‘It is no secret that I have received tobacco sponsorships and still receive and sell tobacco at a nightclub that I co-own and at another music event, which I arrange. I also play at music venues, which sell tobacco. Tobacco is a natural part of the food chain; it is not something you think about, it is something you budget for.’* (Strøm worker)

A few interviewees noted that the smoking behaviour of performing artists and role models in general is an important factor in the smoking initiation and smoking behaviour of young people.

*‘When artists smoke while they perform, then I feel like smoking. I take it almost as an invitation to smoke. I attended a concert at [Danish concert-venue] and everyone smoked. It seemed like the security guards gave up trying to stop it.’* (Strøm worker)

Most interviewees, however, found that role models from the music scene do not influence their own smoking behaviour or that of young people. They also did not consider their own smoking to have an influence on young people.

*‘It’s a safe space when you are on the stage and then I smoke if I want to. I don’t give a shit if you are not allowed to smoke. It’s a part of going out and the first thing I think about when I drink a beer.’* (Strøm worker)

### End exposure of tobacco products and eliminate tobacco industry sponsorship in all Strøm events

The partnership developed an official code of conduct to end exposure to tobacco at all Strøm events:

- There will be no exposure of tobacco products, smoking or any kind of symbol relative to smoking in the press, promotion and communication material developed and distributed by Strøm;
- Strøm staff members should avoid appearing in Strøm related contexts while smoking;
- Strøm will not receive any kind of sponsorships from the tobacco industry or tobacco related organisations and;
- There will be no sale of tobacco products or e-cigarettes at Strøm events.

The code was published on the Strøm website and displayed in print at Strøm’s office. After implementing the code, smoking and tobacco products were no longer included in the visual material related to Strøm.

Through the Strøm partnership and the various tobacco-free initiatives implemented at Strøm events, the visibility of smoking has significantly decreased in the context of the festival. Through the interviews, it was clear that the Strøm code of conduct has increased awareness among Strøm employees of the potential influence of their smoking behaviour on adolescents.

The partnership entailed financial support to develop and run the initiatives. Eliminating the sale of tobacco did not pose a financial constraint to Strøm, which might be due to the festival being less dependent on revenue from tobacco sales as many Strøm events are located in urban spaces where tobacco is available from nearby convenience stores and supermarkets.

**Figure:**
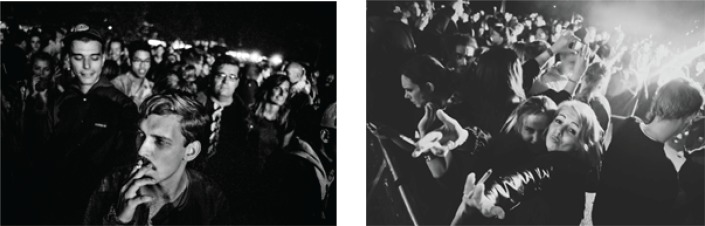
Official press photos of the audience at Strøm Festival 2014 (before implementation of code)

**Figure:**
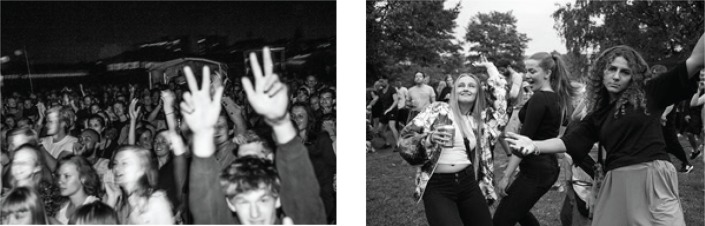
Official press photos of the audience at Strøm Festival 2015 (after implementation of the code)

### Survey among external network

The third initiative consisted of a survey among stakeholders in the Danish music industry. Stakeholders included bookers, artists, journalists, stakeholders from record companies, industry associations and festivals. In all, 316 persons were contacted and 161 persons (51%) responded to the survey. The aim of the survey was not to gain a fully representative data set but rather to outline the main trends and the potential for adopting further tobacco-free initiatives in the music environment. A secondary objective was to inspire reflection among stakeholders on the relationship between tobacco and music events, while building awareness of the possibility to change existing practices.

The survey results suggest positive attitudes towards adopting tobacco-free initiatives to change the norms of tobacco involvement in the music industry. However, the main challenges related to financial sustainability without tobacco industry funding remain substantial.

Interviewees reflected on the close links between tobacco and music industry and some questioned the morality of these relations. The stakeholders displayed considerable variation in their will to initiate similar initiatives to those of Strøm. Some would like to continue the initiatives in other parts of the music business, while others found them too radical.

Several respondents considered tobacco industry funding a normal and essential part of their business strategies and pointed to the financial challenges associated with leaving behind tobacco industry funding and the concern for a decrease in the alcohol sale following restrictions in the sale of tobacco.

Many respondents were, however, very positive or positive (81%) towards the Strøm code of conduct and the initiatives taken to limit exposure of tobacco and smoking.

Twenty-four respondents to the survey represent organizations and venues, which could potentially implement tobacco-free initiatives similar to those of Strøm. Of the 24 respondents, 51% expressed that they were very interested or interested to some extent in implementing similar tobacco-free initiatives.

The sale of tobacco products and the acceptance of tobacco sponsorships at musical events seemed to be motivated mainly by financial incentives. The respondents feared both financial insecurity without tobacco funding and negative response from the audience following increased focus on tobacco control.

On the other hand, the high level of support from music organisations to take on tobacco-free initiatives (51%) points to a potential for expanding the tobacco-free initiatives. Expansion to other organisations would, however, require alternate sources of financing to replace tobacco related income.

## conclusions

The Danish music scene is a valued arena for the tobacco industry’s promotion of smoking to young people. In general, there is low awareness among stakeholders in the music industry both of the powerful role of the tobacco industry in its promotion strategies and of their own role in the indirect tobacco promotion to young people. In order to denormalise tobacco in the music scene, a vital first step is to create awareness among the stakeholders. In the Danish musical festival Strøm, a code of conduct to cease indirect tobacco promotion and tobacco sponsorships was a feasible measure towards this goal. To further disseminate the code and denormalise the presence of tobacco in the music scene, it is important to counter both the financial incentives for festivals to promote tobacco and the fear of negative audience response. We have provided an example of how to initiate the denormalisation of tobacco at the music scene and highlighted the importance of raising awareness among artists and other stakeholders from the music scene and the potential for changing the perception of tobacco as an integral part of music culture.
